# Penile Hemodynamic Response to Phosphodiesterase Type V Inhibitors after Cavernosal Sparing Inflatable Penile Prosthesis Implantation: A Prospective Randomized Open-Blinded End-Point (PROBE) Study

**DOI:** 10.1155/2021/5548494

**Published:** 2021-06-28

**Authors:** Adham Zaazaa, Michaela Bayerle-Eder, Ramzy Elnabarawy, Mahmoud Elbitar, Taymour Mostafa

**Affiliations:** ^1^Department of Andrology, Sexology & STIs, Faculty of Medicine, Cairo University, Cairo 11562, Egypt; ^2^Department of Endocrinology & Metabolism, Medical University of Vienna, Währinger Gürtel 18–20, Vienna 1090, Austria

## Abstract

Forceful corporal dilatation amidst penile prosthesis implantation may injure cavernosal arteries compromising penile vasculature. In this study, we aimed to compare the conventional and cavernosal sparing techniques regarding cavernosal artery preservation. Overall, 33 patients underwent inflatable penile prosthesis implantation with Coloplast Titan Touch® three-piece inflatable penile implants. 16 patients had conventional implantations with serial vigorous dilatations, while 17 patients were implanted with the cavernosal sparing technique, consisting of a single minimal corporal dilatation after an intraoperative intracavernosal injection (ICI) of Alprostadil. Postoperatively, a penile duplex Doppler ultrasound study was performed. Whenever a cavernosal artery was spared and thus successfully probed, its hemodynamics were studied before and after an oral administration of a phosphodiesterase type 5 inhibitor (PDE5i). A cavernosal artery was successfully probed in 16/17 (94%) of patients in the cavernosal sparing group compared to 5/16 (31%) of patients in the conventional group with a significant statistical difference (*P*=0.001). This demonstrated that the cavernosal sparing technique was superior to the conventional approach in preserving the cavernosal artery (odds ratio 35.2, 95% IC 3.5–344.2; *P*=0.0022). Whenever a cavernosal artery could be probed, its hemodynamic responsiveness was also preserved. This trial is registered with NCT03733860.

## 1. Background

Since the advent of the inflatable penile prosthesis (IPP) in 1973, it has become the surgical gold standard in treating patients with refractory erectile dysfunction (ED) [1, 2]. Three-piece inflatable implants have shown greater satisfaction among patients compared to the semi-rigid rods, due to their better concealability and more natural erections [3]. Patients' satisfaction rates after three-piece inflatable implants have been estimated to be up to 98% and up to 96% among their partners [4].

Corporal dilatation during penile prosthesis implantation procedures can injure the cavernosal arteries. This would compromise the blood supply of the corpora and make them solely dependent on the dorsal and bulbourethral arteries. The postoperative application of tight dressings could then potentially compress these arteries against the underlying cylinders resulting in distal ischemia [5].

Cavernosal arteries also play an indirect role in glandular tumescence. The glans penis is mainly supplied by the dorsal arteries and the terminal branches of the spongiosal arteries. Glandular tumescence is partly caused by compression of the deep dorsal and circumflex veins between the engorged corpora cavernosa, supplied by the cavernosal arteries, and the surrounding tissues [6]. Thus, a lack of glans engorgement might be one of the indirect sequelae of cavernosal artery injury during penile prosthesis implantation. Additionally, postoperative loss of subjective penile tumescence and decreased penile girth often occur due to corporal tissue damage [6]. The erection obtained by means of a penile implant may therefore be perceived as “artificial” when it is uncoupled from physiological tumescence.

Efforts have been made to preserve some postoperative residual erectile function in the form of spontaneous tumescence and increased penile girth during sexual activity, which may play an important role in inciting female arousal [7, 8]. Therefore, PDE5i were prescribed after penile prosthesis implantations to increase patients' and partners' satisfaction by enhancing glans engorgement [9, 10]. In that context, Mulhall et al. [11] pointed out that patient satisfaction was indeed significantly increased with the implant-PDE5i combination compared to the implant alone. Grasso et al. [12] also noted an improvement in PSV in response to PDE5i, up to 6 years postimplantation. Lastly, Zaazaa and Mostafa [13] were able to detect pulsating cavernosal arteries by penile duplex Doppler ultrasound (PDDU), after malleable penile prostheses were implanted using the cavernosal sparing technique. In the present study, we adapted that technique to three-piece inflatable penile implantations in an attempt to spare the cavernosal arteries.

## 2. Materials and Methods

The study protocol was approved by the Institutional Ethical Committee and Review Board and informed consent was taken from all patients before the start of the study. The study took place from March 2019 to December 2020. Eligibility criteria included males over 18 years of age with ED of more than one year, unsatisfied with all types of medical treatment. All patients underwent a preoperative PDDU study, in which penile hemodynamics, peak systolic velocity (PSV), end-diastolic velocities (EDV), and resistivity index (RI) were recorded at baseline and 20 minutes after an ICI of 20 *μ*g Alprostadil. Erection was assessed with the erection hardness grading scale. Only patients with adequate cavernosal artery arterial perfusion (PSV >30 cm/sec), obvious veno-occlusive mechanism disorder (EDV ≥6 cm/sec), and an erectile response ranging from 2 to 3 on the erection hardness grading scale were included [14–19].

Patients excluded from the study were patients who had previously undergone penile surgeries (e.g., hypospadias repair, correction of curvature, previous penile prosthesis implantation, and removal), patients with history of priapism or Peyronie's disease (whether causing a palpable mass and/or curvature or deformity on examination), and patients on organic nitrates and unwilling to retake PDE5i postoperatively.

Forty-two patients met the eligibility criteria. Nine patients were excluded from the study, four patients for choosing a different treatment modality, and five patients for declining to participate. Patients meeting the inclusion criteria were randomized to cavernosal sparing and conventional implantation groups using sealed envelopes. All patients were implanted with Coloplast Titan Touch® three-piece inflatable penile implants (Titan Touch, Coloplast, Minneapolis, MN, USA).

### 2.1. General Operative Considerations

Trichotomy was performed on the table and the skin was cleansed twice with Sterillium® disinfectant (HARTMANN International, Heidenheim Germany) before the application of povidone-iodine 10% (Betadine® Solution, Avrio Health LP, USA). The patient was placed in a supine position under spinal anesthesia and was catheterized with an indwelling foley urinary catheter. A purely scrotal longitudinal incision was chosen over the traditional penoscrotal incision due to its more esthetic healing. Two longitudinal corporotomies were performed, each between two sets of double stay sutures of 2/0 Monocryl. Double suturing (two sutures on each side of the corporotomy) ensured the stay sutures were less liable to tear during dilatation and implantation. Dilatation was deemed adequate when each dilator proximally reached a bone stopping point and distally extended well beyond the coronal sulcus. The lengths of the cylinders were deemed suitable when neither buckling nor a dropped glans were encountered intraoperatively after cylinder inflation. The same stay sutures were used to close the corporotomies.

### 2.2. Hemostatic Considerations

To decrease the occurrence of a postoperative scrotal hematoma, we adopted a 3-step approach to ensure better hemostasis. Before closing the scrotal incision, a 2 ml solution of ephedrine HCl was instilled into the scrotal cavity. A fairly tight wrap was applied to the scrotum to which the patients were advised to continuously apply ice packs for 3 days postoperatively. To avoid any urine soiling the wrap, the foley catheter was left in place until all bandages were removed.

### 2.3. Conventional Implantation

In the conventional surgery group, serial dilations of the corpora were carried out with Hegar dilators ranging from a size 8 up to a size 13 whenever possible. Dilatations were carried out with no prior intraoperative intracavernosal injection. Each dilator was passed twice proximally and twice distally, once in the dorsomedial plane and once in the ventrolateral plane, to ensure maximal dilatation.

### 2.4. Cavernosal Sparing Implantation

Unlike what we did in the group undergoing conventional surgery, patients that underwent cavernosal sparing implantation were intraoperatively injected with 40 *μ*g Alprostadil intracorporeally to achieve intraoperative tumescence. Dilation was carried out with a size 7 Hegar dilator only, aiming at minimally disrupting the cavernous tissue and to avoid severing the cavernosal artery during implantation. It is worth noting that dilating tumescent corpora with engorged cavernous sinusoids was remarkably smoother than dilating through a detumescent penis with contracted cavernous tissue.

### 2.5. Postoperative PDDU Study

PDDU study was performed by a blinded operator, 4 to 6 weeks after implantation. A 7.5 MHz linear ultrasound transducer in superficial mode was used (Shenzhen Mindray Bio-Medical Electronics, Shenzhen, China). The presence of a pulsating cavernosal artery, around the inflated cylinders in the shaft of the penis, was probed. Care was taken not to confuse the dorsal artery pulsations with the cavernosal artery which should typically appear adjacent to the cylinders. If the cavernosal artery was not detected in both corpora, or segments of it detected with no pulsatile blood flow, it was deemed severed during dilatation or implantation. In case a pulsatile cavernosal artery was detected in either corpus, its hemodynamics were recorded both at baseline and after combined pharmacological and manual stimulation.

**Figure 1:**
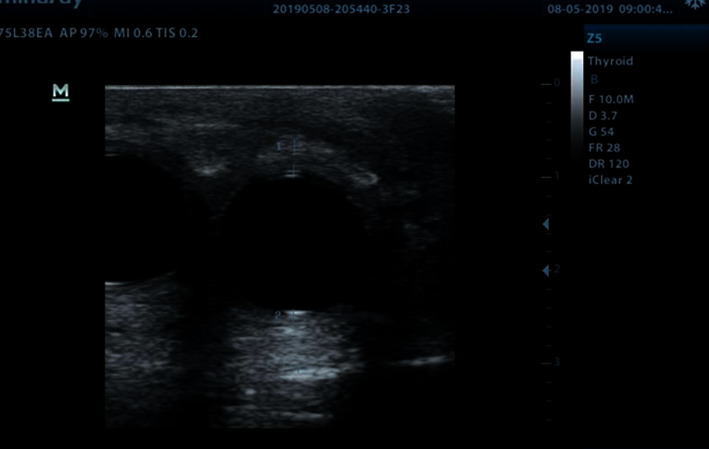
Inflated cylinders. 2D ultrasound image showing the inflated cylinders before penile hemodynamic measurement.

**Figure 2:**
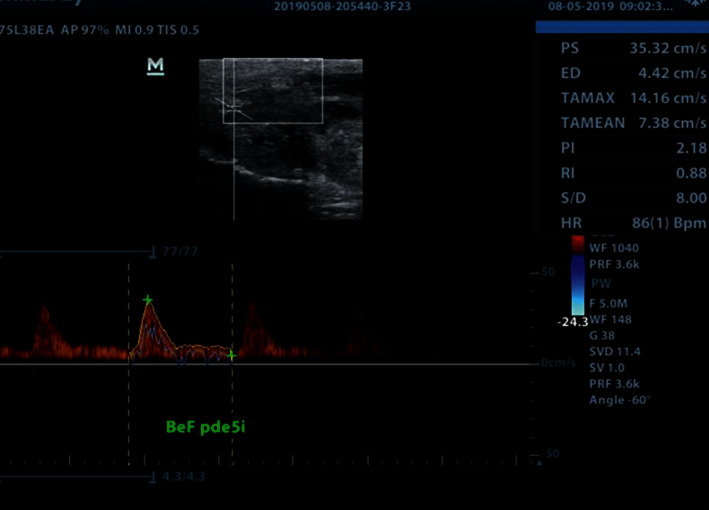
Postoperative baseline hemodynamics. PDDU showing postoperative baseline hemodynamics before administration of PDE5i. Note that Figures 2 and 3 are for the same patient.

**Figure 3:**
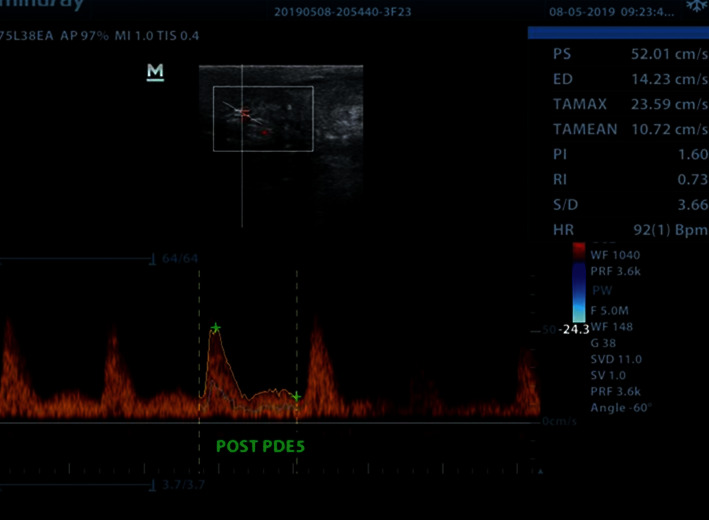
Postoperative post-PDE5i hemodynamics. Postoperative post-PDE5i hemodynamics measurements showing pronounced cavernosal artery waves, recorded 20 minutes after PDE5i administration and manual stimulation. Please note that Figures 2 and 3 are for the same patient.

With the implants inflated **(**Figure 1), PSV, EDV, and RI were recorded at baseline (Figure 2). The patients were then administered a 10 mg orodispersible tablet of Vardenafil hydrochloride (Vivanza, Bayer). After 10 minutes, they were instructed to manually stimulate their penis for about 10 more minutes. PSV, ESV, and RI were recorded once more after sexual stimulation (Figure 3).

### 2.6. Statistical Analysis

Statistical analysis was performed using SPSS version 23 (SPSS Inc, Chicago, IL, USA). Paired *t*-test, ANOVA, and Fisher Exact test were used as statistical tools. *P* value <0.05 was set as statistically significant.

## 3. Results

Overall, 33 patients were randomized into 2 arms (16 patients received conventional implantations and 17 patients received cavernosal sparing implantations). The mean baseline PSV in both the cavernosal sparing and conventional groups at baseline as well as after ICI showed nonsignificant differences (*P*=0.553; *P*=0.314, respectively). Patients in both groups showed veno-occlusive dysfunction with no significant differences between their EDV (*P*=0.561) (Table 1).

### 3.1. Postoperative Measurements

The postoperative detection of at least one pulsating cavernosal artery was achieved in 16/17 (94%) of patients in the cavernosal sparing group compared to 5/16 (31%) of patients in the conventional group (*P* < 0.001). The cavernosal sparing technique was thus superior to the conventional approach in preserving the cavernosal artery (odds ratio 35.2, 95% IC 3.5–344.2; *P*=0.0022). In the cavernosal sparing group, both PSV and EDV increased in response to PDE5i administration (*P* < 0.001; *P*=0.001, respectively). In the conventional group, PSV increased significantly in response to PDE5i administration (*P* < 0.001), while EDV showed nonsignificant change (*P*=0.720) **(**Table 2). Hence, whenever an artery was spared, its PSV hemodynamic response to PDE5i was similarly preserved, whether it was spared by means of the cavernosal sparing technique or during a conventional implantation.

### 3.2. Surgical Complications

One patient in the conventional group exhibited posterior perforation and surgery was resumed with sling sutures applied to the rear tip extender [20]. No perforations were encountered in the cavernosal sparing group. Two patients in the cavernosal sparing group developed a postoperative scrotal hematoma that was treated conservatively. All of these aforementioned complications were of grade I of the Clavien–Dindo classification [21].

## 4. Discussion

One of the key steps of conventional penile prosthesis implantations is serially dilating the corpora with dilators of incrementally increasing sizes. This step might inevitably cause cavernous tissue damage evident by the ubiquitous scarring in redo cases. Therefore, many attempts have been made to minimize corporal tissue injury during this step.

In their randomized controlled trial on 100 patients, Moncada et al. [22] suggested omitting the dilatation step altogether in virgin cases. This not only lessened postoperative pain due to less tissue damage but also preserved some residual erectile function, enabling patients to score higher on questions 1 to 3 of the International Index of Erectile Function (IIEF) questionnaire.

Zaazaa and Mostafa [13] proposed an additional cavernous tissue-sparing technique consisting of minimal dilatation after pharmacologically induced tumescence. In that randomized controlled trial on 92 patients, the cavernous tissue-sparing technique yielded a higher incidence of postoperative subjectively reported tumescence. Postoperative ultrasound imaging showed superior radial cavernous tissue thickness translating into a significantly higher postoperative penile girth.

In this context, tumescence, with its subsequent increased penile girth, not only is important for subjective arousal but also enhances the partner's excitement in terms of the perception of “being desired” [7, 8, 23]. Another advantage of preserving cavernous tissue would be the preservation of postoperative tumescence hence decreasing the likelihood of a “floppy glans” thus enabling easier vaginal penetration [24].

In the current study, we applied the cavernosal sparing technique to inflatable penile prosthesis implantation. We then studied residual erectile function in the form of cavernosal artery responsiveness to PDE5i. The preservation of a cavernosal artery and hence its PDE5i responsiveness was noted in 16/17 (94%) of patients in the cavernosal sparing group versus 5/16 (31%) of patients in the conventional group with significant difference.

Intraoperative tumescence and minimal dilatation are the two principal steps in the cavernosal sparing technique. In the current series, the cavernosal artery was spared in 5/16 (31%) of patients in the conventional group despite serial dilatations. It could be postulated that this might be due to suboptimal dilatations and/or accidental intraoperative tumescence. Welti and Brodsky [25] as well as Baltogiannis et al. [26] have reported intraoperative erections with an incidence of up to 2.4%, suggesting that intraoperative tumescence, not full erection, might be of even higher incidence, but would pass unnoticed or unreported. Inline, Manning et al. [27] reported spontaneous tumescence after 3-piece prosthesis implantation in 53% of their cases. Another case report by Yildirim et al. [28] reported the occurrence of a complete rigid erection without inflating the implant cylinders.

Other than spontaneous tumescence, PDE5i-induced tumescence was also noted after penile prosthesis implantation. In their study, Mulhall et al. [11] endorsed prescribing up to 100 mg sildenafil citrate after penile prosthesis implantations to enhance glans engorgement, with a significantly better IIEF score achieved in those patients compared to patients not receiving PDE5i. In the current study, penile hemodynamic responsiveness to PDE5i was used as our outcome measure instead of the IIEF score.

In another series of 12 patients with Peyronie's disease undergoing malleable penile implantations for correction of acquired penile curvatures, Grasso et al. [12] demonstrated improved cavernous tissue thickness from 5 mm to 9 mm and increased PSV from 7.5 cm/s to 16.5 cm/s both at baseline and then after administration of 50  mg sildenafil citrate, up to 6 years postimplantation. This postoperative increase in PSV was reproduced in our study whenever a cavernosal artery could be probed.

In our experience, corporal dilatation was found to be smoother with intraoperative tumescence, as the dilator would glide in a path of least resistance, through blood-filled sinusoidal spaces, as opposed to being pushed through the contracted cavernous tissue of a flaccid penis [29]. Additionally, intraoperative tumescence enhanced the assessment of penile size and shape, allowed for a better grip during surgical manipulations, and enabled easier installations of the paracorporotomy stay sutures.

In response to the omnipresent question among implant surgeons: to dilate or not to dilate? It could be assumed from the current series that minimal dilatation should suffice in virgin cases. Serial dilatations can be reserved for cases with corporal fibrosis and scarring, such as Peyronie's disease, neglected priapism, or redo cases.

Previously reported advantages of the cavernosal sparing technique include preserving subjectively reported tumescence [13], optimizing postimplantation penile dimensions [30], and theoretically nonetheless, enhancing postoperative antibiotics reach to the plane around the implants [31]. This study proposes the cavernosal sparing implantation technique as a means of further enhancing postoperative PDE5i hemodynamic responsiveness, after inflatable penile prosthesis implantations.

Still, the current study's main limitations are the lack of placebo control and validated questionnaires such as the Erectile Dysfunction Inventory of Treatment Satisfaction (EDITS) questionnaire [32] or IIEF score [33]. Further studies are also needed to correlate between the sonographic hemodynamic responsiveness and clinical manifestations such as glans engorgement and penile tumescence.

## 5. Conclusions

The present study gives an ultrasonographic rationale for the use of PDE5i after inflatable penile prosthesis implantations. Given the observed results, we recommend the use of the cavernosal sparing technique in virgin inflatable penile prosthesis implantations to potentially enhance the postoperative response to pharmacological adjuvant therapies.

## Figures and Tables

**Table 1:** Patients' characteristics and preoperative baseline measurements (mean ± SD, range).

	Cavernosal sparing group (*n* = 17)	Conventional group (*n* = 16)	*P*
Age (years)	47.4 ± 12.3 (30.0–69.0)	54.3 ± 11.8 (36.0–71.0)	0.1106
BMI (kg/m^2^)	27.2 ± 1.3 (25.0–29.7)	26.8 ± 1.1 (25.2–29.1)	0.3489
ED duration (years)	4.7 ± 2.1 (2.0–8.0)	5.1 ± 2.4 (2.0–9.0)	0.6134
Basal PSV (cm/s)	17.7 ± 5.8 (10–30)	19.1 ± 4.1 (11.3–27.7)	0.4321
PSV (cm/s) after ICI	49.9 ± 13.2 (30.9–80.0)	45.6 ± 10.9 (32.23–71.7)	0.3516
Basal EDV (cm/s)	0.1 ± 0.4 (0.0–1.4)	0 (0)	—
EDV (cm/s) after ICI	12.6 ± 4.0 (6.1–20.9)	11.8 ± 3.6 (6.1–20.1)	0.5512

BMI: body mass index; PSV: peak systolic velocity; EDV: end-diastolic velocity; ICI: intracavernosal injection.

**Table 2:** Postoperative measurements of spared cavernosal artery (mean ± SD, range).

	Cavernosal sparing group (*n* = 16)	Conventional group (*n* = 5)	*P*
PSV baseline (cm/s)	33.5 ± 7.6 (17.7–43.2)	28.4 ± 7.8 (18.1–40.1)	0.0708
PSV (cm/s) after PDE5i	56.4 ± 11.5 (36.8–83.4)	51.2 ± 5.7 (45.5–60.2)	0.1156
*P*	<0.001	<0.001	

EDV baseline (cm/s)	5.7 ± 4.7 (0.0–13.7)	11.6 ± 7.4 (0.0–26.9)	0.1708
EDV (cm/s) after PDE5i	8.4 ± 5.5 (2.3–15.2)	9.4 ± 2.3 (7.3–13.1)	0.7173
*P*	0.001	0.720	

Paired *t-*test. PSV: peak systolic velocity; EDV: end-diastolic velocity; PDE5i: phosphodiesterase type 5 inhibitor.

## Data Availability

The datasets collected and/or analyzed during the current study are available from the corresponding author on reasonable request.

## Abbreviations

ED: Erectile dysfunction
EDITS: Erectile dysfunction inventory of treatment satisfaction
EDV: End-diastolic velocity
ICI: Intracavernosal injection
IIEF: International index of erectile function
IPP: Inflatable penile prosthesis
PDDU: Penile duplex Doppler ultrasound
PDE5i: Phosphodiesterase type 5 inhibitors
PROBE: Prospective randomized open-blinded end-point
PSV: Peak systolic velocity
RI: Resistivity index.
